# Sepsis in a Seropositive Pregnant Woman With Early Preterm Labor Pains: A Case Study of a Near Miss

**DOI:** 10.7759/cureus.29815

**Published:** 2022-10-01

**Authors:** Aarshika Singh, Mayur S Raka, Ronak H Rukhiyana, Ooha Thadiboina

**Affiliations:** 1 Obstetrics and Gynaecology, Jawaharlal Nehru Medical College, Datta Meghe Institute of Medical Sciences, Wardha, IND

**Keywords:** sepsis, antiretroviral therapy, preterm delivery, pregnancy, hiv

## Abstract

HIV is linked to a higher risk of preterm delivery in pregnant women. A systemic response to HIV virus can lead to foetus death along with patient death. Mortality is reduced in pregnant females and neonates by some interventions done carefully like antiretroviral therapy and prophylaxis, careful delivery methods, and monitoring of safe breastfeeding. Precautions are also used to decrease the mother-to-child transmission of HIV. An HIV-positive pregnant woman with sepsis is presented here to highlight the management of sepsis and labour. An HIV-positive primigravida on regular tenofovir, lamivudine, and efavirenz (TLE) regimen presented at 29 weeks and five days of her pregnancy to our outpatient department (OPD) with complaints of thick pus-like discharge and fever from seven to eight days. To manage it, labour was augmented by oxytocin in drip. Under all aseptic precautions, a breech 1.1kg male baby was delivered three hours later. Post-delivery status of the patient was uneventful except for two episodes of fever for two days serially on day five and day six. Both mother and the baby were discharged after 43 days of in-ward stay, both symptomatically alright. The mother was advised to continue antiretroviral therapy and get six monthly CD-4 (cluster of differentiation 4) counts for review and the baby was to be kept on top feeds till six months of age at the request of the patient. Keeping the following guidelines in mind, a multidisciplinary approach works best for such cases of HIV-infected mothers. However, it is necessary to individualise each patient.

## Introduction

HIV is linked to a higher risk of preterm delivery (premature rupture of membranes), low birth weight, and intrauterine death. Anti-retroviral treatment and prophylaxis, careful delivery methods, and monitoring of safe breastfeeding are some of the interventions used to decrease the mother-to-child transmission of HIV. High viral loads, low CD4 (cluster of differentiation 4) levels, advanced disease, and the occurrence of prolonged labour and deliveries, particularly when exacerbated by chorioamnionitis, all enhance the risk of HIV transmission to the foetus [1]. Elective caesarean section is best done at 38 weeks, especially when the viral load is greater than 1000cc/ml. The WHO criteria for initiation of antiretroviral therapy in adults are as follows: in stage IV, stage III at CD4<350cc/ml, and stage I and II when CD4 is less than 200cc/ml [2]. CD4 levels increase by greater than 50cc/ml at four to eight weeks in response to highly active antiretroviral therapy and by an additional 50 to 100 per year thereafter [3]. Counselling of the family is necessary when the patient is started on antiretroviral therapy [4]. A case of an HIV-positive pregnant woman with sepsis is presented here to highlight the management of sepsis and labour in an HIV-positive patient.

## Case presentation

**Figure 1:**
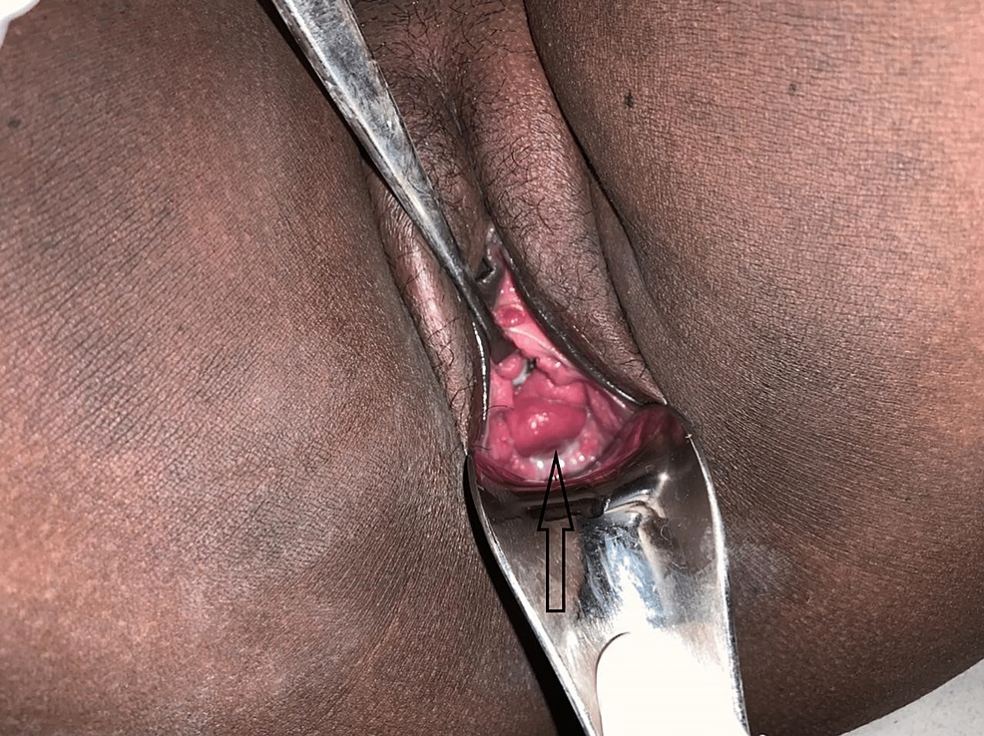
Discharge coming from vagina on per speculum

A 35-year-old primigravida whose HIV-positive status has been known since 2015 on regular tenofovir, lamivudine, and efavirenz (TLE) regimen, married to a man of unknown HIV status (second marriage), presented at 29 weeks and five days of her pregnancy to our outpatient department (OPD) with complaints of thick purulent discharge (Figure 1) and fever since seven to eight days ago. On examination, the patient was conscious, oriented and febrile (100.2^0 ^F), had tachycardia (132 bpm), blood pressure (80/50 mmhg), and ongoing contractions per abdomen. On per speculum and per vaginum examination, there were copious amounts of thick yellowish white purulent non-offensive discharge coming out of her vagina. The cervix was 2cm dilated and 50-60% effaced and the station of the presenting part (foot) was coming to -1; the membranes were absent (premature preterm rupture of membranes). At this point, a working diagnosis of septic shock with premature preterm rupture of membranes was made and a multidisciplinary approach to management was initiated. After primary resuscitation, initial blood samples were drawn, and a high vaginal swab was taken for culture and sensitivity. The lab investigations are mentioned in Table 1.

**Table 1:** Lab report

Parameter	Value	Normal limits
Haemoglobin	10.8 gm/dl	11.5-15 gm/dl
Total leucocyte count	26000 Cu.mm	4000-11000 Cu.mm
C-reactive protein	80.24 gm/dl	< 6 gm/dl
Platelets	3.09 lakhs/Cu.mm	1.5 to 4.5 lakhs/Cu.mm
Lactate dehydrogenase	578 IU/L	320-780IU/L
Blood sugar	129 mg/dl	<140 mg/dl

The liver and renal function tests, and coagulation profile were within limits. The most recent CD4 counts (2 months ago) were 1049 cc/ml. An emergency physician consult was sought for sepsis and to prevent disseminated intravascular coagulation given the high-risk nature of the pregnancy. Higher injectable antibiotics (piperacillin-tazobactam, meropenem, and metronidazole) were started and she was advised to continue with her ongoing antiretroviral therapy: TLE regimen. Urgent ultrasonography was done after stabilising the patient, which revealed a single live intrauterine foetus (SLIUF) of 28 weeks and one day an estimated foetal weight (EFW) of 1205gm, double loop of cord around the neck, and severe oligohydramnios with liquor index 1. Cervical length was given at approximately 3cm. Labour was augmented by oxytocin in drip. Under all aseptic precautions, a breech 1.1-kilogram male baby was delivered three hours later. The baby cried immediately, and delayed cord clamping was done. The baby was resuscitated following standard neonatal intensive care unit protocol with an APGAR (Appearance, Pulse, Grimace, Activity and Respiration) score of 8/10; 9/10.

**Figure 2:**
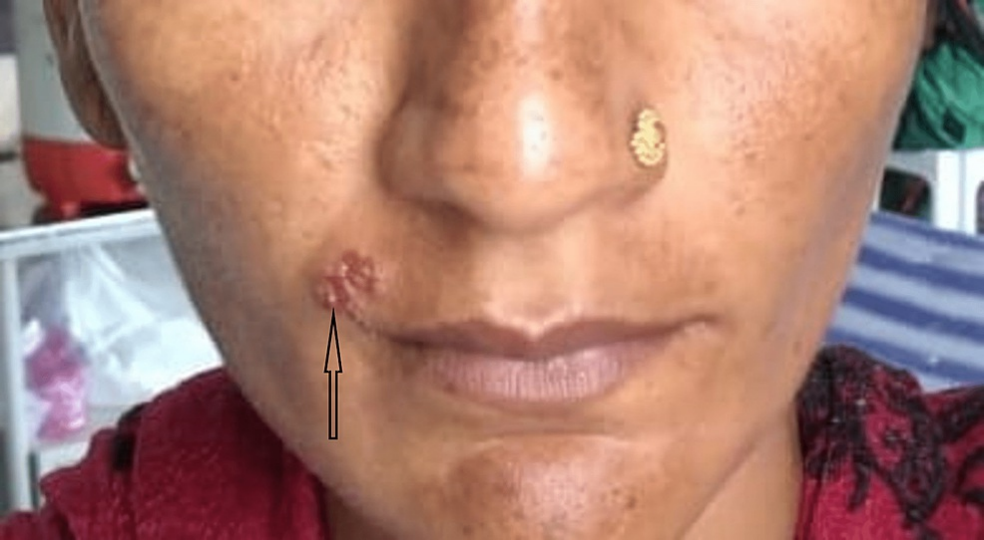
Herpes labialis near mouth

Post-delivery status of the patient was uneventful except for two episodes of fever for two days serially on day five and day six. On examination, the patient had breast engorgement as she had declined to breastfeed the baby when given the option of exclusive breastfeeding. Lactation was suppressed by tab cabergoline and B-6. Injectable antibiotics were continued till day seven and shifted to oral, amoxicillin-clavulanate, 625 mg thrice a day for the next five days with strict temperature monitoring. Blood counts were also monitored, with the total leukocytes count coming down from 26,000 (on admission) to 10,500, to 7200, and finally to 4100 at the time of discharge. The patient developed lesions near the mouth around the lips on post-delivery day three following which we sought the opinion of a dermatologist. According to them, the nodules were suggestive of herpetic lesions (Figure 2). For the same, they prescribed oral acyclovir for two weeks and the symptoms subsided. Herpes labialis is a common complication of HIV [5] due to the immunocompromised nature of the diseased individuals. Antiretroviral therapy was continued throughout: TLE regimen (300/300/600mg; combined dose once a day daily).

The baby was admitted to the neonatal intensive care unit for one month as it was extremely preterm and had a very low birth weight. He was kept on oxygen gas (O2) by continuous positive airway pressure and later on by nasal prongs. Intravenous fluids and injectable piperacillin-tazobactam and amikacin were also administered. Syrup nevirapine was administered as per protocol. His septic screen was sent twice during his neonatal intensive care unit stay, which were both normal. Standard immunisation for the baby was done: bacille Calmette-Guerin (BCG), oral polio vaccine, and hepatitis B vaccine. Syrup nevirapine dose was completed in the ward as per protocol. Both mother and the baby were discharged after 43 days of in-ward stay when both were symptomatically alright. The mother was advised to continue antiretroviral therapy and get six monthly CD4 counts for review. The baby was to be kept on top feeds till six months of age and regular fortnightly paediatric check-ups for weight gain and immune status or SOS (si opus sit). Contraceptive counselling was emphatically done.

## Discussion

Premature labour, foetal infection, and preterm birth are all linked to pregnancy complications such as sepsis and septic shock. The development of sepsis in pregnancy can be subtle and individuals often seem normal at first before quickly worsening and dying from septic shock, multiple organ dysfunction syndromes, and even fatality. Prompt screening, timely diagnosis of the infection source, and specific therapy improve survival and outcome in severe sepsis and septic shock in pregnancy. This could be accomplished by devising a methodical algorithm that includes early time-sensitive approaches such as fluid intake (20 ml/kg of normal saline over the first hour), empirical injectable antimicrobials (gentamicin, clindamycin and penicillin) within 60 minutes of diagnosis, vitals’ tracking, and involving infectious disease experts and intensivists who are knowledgeable about pregnancy and related physiological changes [6]. To locate the septic origin, a complete physical assessment, radiological modalities, or prophylactic exploratory laparotomy are recommended. Patients may keep on deteriorating even with effective antimicrobial therapy until septic foci (e.g, pus collection, necrotic tissue) is removed surgically. In case of antepartum sepsis or septic shock, the choice to deliver must be made based on gestational age, maternal state, and foetal status. The natural temptation is to induce emergent delivery because of the precarious foetal health, but it is necessary to stabilise the mother first since this will also improve foetal outcome. Preoperative skin preparations and prophylactic antibiotic therapy as well as proper vaccination can be predicted to lower the severity of sepsis and septic shock and methods can include preoperative skin preparations and prophylactic antibiotic medication [7]. HIV has a few effects on the first line of defence. To begin, epithelial barrier abnormalities are prevalent and widely documented in the gut. Throughout acute infection, HIV induces barrier abnormalities in the gut, which persist during chronic infection, allowing invasive infections by intestinal bacteria such as non-typhoidal salmonella. In addition, microbial compounds that enter the bloodstream promote chronic immunological activation and weariness [8].

In HIV patients, bacterial sepsis is a leading cause of morbidity and mortality. HIV increases susceptibility to invasive infections and alters the pathophysiology of sepsis, which is characterised by pre-existing immune system activity and depletion [9]. We look at the impact of HIV on several immunological responses linked to bacterial sepsis, as well as potential mechanisms behind the increased risk of invasive bacterial infections. Pattern recognition receptors and innate cellular responses, cytokines, lymphocytes, coagulation and the complement system are among the topics that are being studied [10]. Increased vulnerability to infection is caused by various variables, which can lead to a dysregulated immune response in HIV patients during a septic episode. The immune system abnormalities caused by HIV vary depending on the stage of infection and are only partially repaired by combination antiretroviral therapy. Because many pathogenic processes in HIV and sepsis overlap, immunomodulatory therapies for sepsis that are now being developed may be especially advantageous to individuals with HIV coinfection. The WHO advised that PMCT (prevention of mother-to-child transmission) treatments provide antiretroviral therapy prophylaxis to newborns from the time of birth to 6-12 weeks. The length of antiretroviral therapy administration to the newborn is determined by the HIV-infected mother's antiretroviral therapy status. For breastfeeding newborns, nevirapine prophylaxis is recommended for the first six weeks. For non-breastfeeding infants, the same antiretroviral therapy prophylaxis is recommended for 4 to 6 weeks.

## Conclusions

In the era of excellent antiretroviral therapy, bacterial sepsis has been a primary cause of death in HIV patients. Even though antiretroviral therapy significantly improves patients' immune function, as seen by a considerable reduction in opportunistic infections, patients still face a high risk of invasive bacterial infection, implying the current and future relevance of this slightly less researched topic. The Indian AIDS control programme has taken considerable steps toward reducing HIV transmission from infected pregnant women to their newborn children. India, on the other hand, is still a long way from attaining 100% HIV mother-to-child transmission elimination. However, more surveillance data is still required to track the progress of the prevention of the mother-to-child transmission programme.
